# Impact of disease activity and treatment of comorbidities on the risk of myocardial infarction in rheumatoid arthritis

**DOI:** 10.1186/s13075-016-1077-z

**Published:** 2016-08-05

**Authors:** Yvette Meissner, Angela Zink, Jörn Kekow, Karin Rockwitz, Anke Liebhaber, Silke Zinke, Kerstin Gerhold, Adrian Richter, Joachim Listing, Anja Strangfeld

**Affiliations:** 1 German Rheumatism Research Centre, Epidemiology Unit, Berlin, Germany; 2 German Rheumatism Research Centre, Epidemiology Unit, and Charité University Medicine Berlin, Berlin, Germany; 3 Otto-von-Guericke University, Magdeburg, Germany; 4 Private Practice, Goslar, Germany; 5 Private Practice, Halle/Saale, Germany; 6 Private Practice, Berlin, Germany

**Keywords:** Myocardial infarction, Cardiovascular disease, Inflammation, Disease activity, Tumour necrosis factor inhibitors, Biologicals

## Abstract

**Background:**

The aim was to estimate the impact of individual risk factors and treatment with various disease-modifying antirheumatic drugs (DMARDs) on the incidence of myocardial infarction (MI) in patients with rheumatoid arthritis (RA).

**Methods:**

We analysed data from 11,285 patients with RA, enrolled in the prospective cohort study RABBIT, at the start of biologic (b) or conventional synthetic (cs) DMARDs. A nested case–control study was conducted, defining patients with MI during follow-up as cases. Cases were matched 1:1 to control patients based on age, sex, year of enrolment and five cardiovascular (CV) comorbidities. Generalized linear models were applied (Poisson regression with a random component, conditional logistic regression).

**Results:**

In total, 112 patients developed an MI during follow-up. At baseline, during the first 6 months of follow-up and prior to the MI, inflammation markers (erythrocyte sedimentation rate (ESR) and C-reactive protein (CRP)) but not 28-joint-count disease activity score (DAS28) were significantly higher in MI cases compared to matched controls and the remaining cohort. Baseline treatment with DMARDs was similar across all groups. During follow-up bDMARD treatment was significantly more often discontinued or switched in MI cases. CV comorbidities were significantly less often treated in MI cases vs. matched controls (36 % vs. 17 %, *p* < 0.01). In the adjusted regression model, we found a strong association between higher CRP and MI (OR for log-transformed CRP at follow-up: 1.47, 95 % CI 1.00; 2.16). Furthermore, treatment with prednisone ≥10 mg/day (OR 1.93, 95 % CI 0.57; 5.85), TNF inhibitors (OR 0.91, 95 % CI 0.40; 2.10) or other bDMARDs (OR 0.85, 95 % CI 0.27; 2.72) was not associated with higher MI risk.

**Conclusions:**

CRP was associated with risk of MI. Our results underline the importance of tight disease control taking not only global disease activity, but also CRP as an individual marker into account. It seems irrelevant with which class of (biologic or conventional) DMARD effective control of disease activity is achieved. However, in some patients the available treatment options were insufficient or insufficiently used - regarding DMARDs to treat RA as well as regarding the treatment of CV comorbidities.

**Electronic supplementary material:**

The online version of this article (doi:10.1186/s13075-016-1077-z) contains supplementary material, which is available to authorized users.

## Background

In rheumatoid arthritis (RA), increased morbidity and mortality due to myocardial infarctions (MI) cannot entirely explained by traditional cardiovascular (CV) risk factors [1–3]. There is evidence that the rheumatic disease itself contributes to the risk of CV events [1, 4–6], with inflammation as the link between RA and CV disease (CVD). Some of the pivotal pro-inflammatory mediators, including the cytokines tumour necrosis factor (TNF), interleukin 1 (IL-1) and interleukin 6 (IL-6) [7, 8], as well as the acute-phase reactant C-reactive protein (CRP), are involved in atherogenesis and eventually in the development of coronary artery diseases like MI [9].

A recent meta-analysis of studies investigating single nucleotide polymorphisms (SNPs) hypothesized a causal role of the IL6R-gene signalling via the inflammatory markers CRP and fibrinogen in the development of coronary heart disease (CHD) [10]. The awareness of even relatively low levels of CRP as a risk factor for MI has increased in the rheumatologic community. A few studies of MI in RA examined prospectively collected CRP [11–15]. Nevertheless, other studies have identified erythrocyte sedimentation rate (ESR) as a relevant inflammation marker in CVD [16–19]. Similarly, high disease activity measured by the composite score based on 28 joints (DAS28) is discussed to have an important influence on the risk of MI [17, 19]. The European League Against Rheumatism (EULAR) recommendations for CV risk management require “adequate control of disease activity” [20]. However, global disease activity might not be sensitive enough in patients at increased risk of MI. Therefore, the question remains whether CRP and/or ESR should be taken into account as additional targets in a treat-to-target approach. Randomised clinical trials are unable to answer this question due to the long latency to the outcome of MI, the restricted follow-up time and the exclusion of patients with major CVD. Observational studies, on the other hand, should be suitable to investigate risk factors for MI. However, comparisons between patients with RA who develop MI and the rest of the cohort are difficult to interpret in observational studies, due to significant differences in age, sex and CV comorbidities [12, 19].

To control for these confounding factors, a few studies applied a matched case-control design [21, 22], but the results are conflicting. Radovits et al. could neither confirm CRP nor DAS28 as risk factors for MI [22], whereas Mantel et al. observed significantly elevated ESR, CRP and DAS28 in cases compared to controls [21]. These contradictory results may be caused by sparse matching procedures: matching for disease duration only [22] and matching for sex, year of RA diagnosis and rheumatologic unit [21].

We pursued two aims with this study: First, to show the influence of risk factors, especially the effect of inflammation, on the incidence of MI in patients with RA. Second, we were interested in the impact of treatment: (1) the treatment of RA with disease-modifying antirheumatic drugs (DMARDs) and concomitant glucocorticoids and (2) the treatment of CV comorbidities. To preclude distorting effects we applied a case–control study with an extended matching algorithm comprising traditional CV risk factors such as age, sex and CV comorbidities.

## Methods

### Data source

Data from the German biologics register Rheumatoid Arthritis: Observation of Biologic Therapy (RABBIT) were used. RABBIT is an ongoing observational cohort study in which patients are included at the start of treatment with a biologic (b)DMARD or a conventional synthetic (cs)DMARD after failure of at least one prior csDMARD [23, 24]. In brief, once enrolled, patients stay in the cohort for at least the next 5 (if possible, 10) years. At regular predefined times (0, 3 and 6 months, and then every 6 months) rheumatologists complete assessment forms at clinical routine visits capturing current clinical status, treatment and all adverse events that have occurred since the last follow-up. Additionally, weight, height as well as existing comorbidities and their treatment are assessed at baseline. At all follow-up visits patients report their global health status using numerical rating scales and their disability by the Hannover Functional Status Questionnaire (FFbH), in which 100 % indicates full functional capacity [25]. Smoking habits are stated at baseline. The study protocol of RABBIT was approved by the ethics committee of the Charité University Medicine Berlin.

### Study design and matching algorithm

We performed a nested case–control analysis based on exact matching where each case was randomly matched to one control patient from the same original cohort. Matching criteria were sex, age at baseline (±3 years) and CV comorbidity at baseline (hypertension, CHD, heart failure, prior cerebrovascular event and hyperlipoproteinaemia). To ensure similar availability of treatment options for each case–control pair, the year of inclusion into RABBIT (±2 years) was also added as matching criteria. Eligible controls had to to be still under observation and without a CV event at follow-up prior to the index date of the corresponding case (calendar date of the MI).

### Case definition

Cases were defined as patients observed in RABBIT with an MI as the first CV event after enrolment up to October 2013. The case definition included the following reported diagnoses: MI (acute, silent or not otherwise specified), ST segment elevation MI, non ST segment elevation MI and anterior or posterior wall infarction. For all reported MIs supplemental information on clinical symptoms, cardiac biomarkers, electrocardiographic changes and imaging results were requested on standardized forms from the rheumatologist. If available, hospital discharge letters and death certificates were reviewed. Individual patients were eligible as a case if their first ever MI had occurred prior to enrolment in RABBIT. These events are possibly subsumed in the comorbidity defined as CHD.

### Validation of cases and controls

In a subgroup of patients (MIs reported to RABBIT until October 2011 and their matching controls, n_pairs_ = 75), on-site visits were performed to revalidate CV events and to verify the control status of the corresponding controls. During these on-site visits, the entire patient records or electronic patient files were reviewed. This comprised inpatient and outpatient records, laboratory results and hospital discharge letters. A CV event listed in the patient record during follow-up, which had not been previously reported to RABBIT, was considered as an event for the analysis. If this event was an MI and had occurred in a control patient, this patient was re-categorized as a case patient and a new control patient was matched for that case. After data collection in the rheumatologic units, the diagnoses of all reviewed patients were validated in a blinded process by a physician (KG) to verify the case and control status. Only confirmed events were included.

### DMARD exposure and concomitant treatment

Data on DMARD treatment are captured in RABBIT at every follow-up time point, and include agent, dose, frequency of administration and start/stop-dates. Concomitant treatment with oral glucocorticoids including dosage in prednisone-equivalents, nonsteroidal anti-inflammatory drugs (NSAIDs) and inhibitors of cyclooxygenase-2 (COX-2) are prospectively collected. In addition, rheumatologists report comorbidities and their medical treatment at baseline. Patients with hypertension, CHD, heart failure or hyperlipoproteinaemia, but without respective treatment, were labelled as having no CV treatment.

For the analysis, bDMARDs were categorized into (1) TNF inhibitor (TNFi) (adalimumab, certolizumab, etanercept, golimumab and infliximab), (2) other bDMARDs (abatacept, anakinra, rituximab and tocilizumab) and (3) csDMARDs. In groups (1) and (2) combination with csDMARD treatment was possible; group (3) was exclusively treated with one or more csDMARDs. We applied two different definitions of bDMARD exposure: to examine treatment changes in the use of bDMARDs and to determine length and frequency of bDMARD episodes we considered the first missed dose or the switch between bDMARDs as discontinuation. In contrast, in the multivariable analysis of the influence of RA treatments on the risk of MI we considered patients as being exposed to a certain DMARD class or glucocorticoid if at least one dose of the drug was prescribed within the last 6 months prior to the MI/index date.

### Statistical analysis

For baseline comparison of MI cases and the remainder of the RABBIT cohort we used the *t* test and Chi-squared test. Comparisons in the matched case–control design were drawn using the paired *t* test or McNemar's test. CRP, ESR and DAS28 were analysed at different times: at baseline, within the first 6 months after enrolment and up to 18 months before the MI/index date. Persistence with enrolment therapy was investigated using Kaplan-Meier estimates. In addition, we were interested in the cumulative number of treatment changes (sequence of DMARD episodes). The switch from a csDMARD to a bDMARD or the reverse and any switch between bDMARDs were counted as treatment changes and were used to calculate treatment episodes. We assumed that the number of switches follow a Poisson distribution and applied a generalized linear mixed model with a random component for the matched case–control design.

Multiple conditional logistic regression analysis was applied to investigate the impact of risk factors on the likelihood of developing an MI (cases vs. controls). The regression model was additionally adjusted for non-matching criteria: CRP, smoking, diabetes and insufficient treatment of underlying CVD. CRP was included as reported values within 6 months prior to the MI/index date (analysis I) and as the average of all reported values from baseline until the MI/index (analysis II). Due to the skewed distribution of CRP values, log-transformed CRP values (logCRP) were calculated. A sub-analysis was applied, excluding patients with a reported CHD at baseline (N_pairs_ for the analysis = 77).

The most frequently missing data among case–control pairs were on patient-reported smoking status (25/224, 11.2 %) at baseline. In subsequent analyses these patients were considered in a separate category (unknown smoking status) and not excluded. Missing data on ESR (CRP) were less frequent: 1.4 % (0 %) at baseline and during follow-up 9.5 % (8.1 %) at most in case–control pairs. In the 6 months prior to MI, values of CRP were not available for seven pairs (six (5.4 %) cases and one (0.9 %) control). For the analysis of the course of disease activity we applied multiple imputations (n_Imputation_ = 5) of missing values. In conditional logistic regression we considered only pairs with observed values of ESR (CRP).

*P* values <0.05 were regarded as statistically significant without adjustment for multiple testing in univariate comparisons. The matching was applied using the R-package Optmatch of the freely available software R [26]. All other analyses were applied using the Statistical Analysis System (SAS) version 9.4.

## Results

**Fig. 1:**
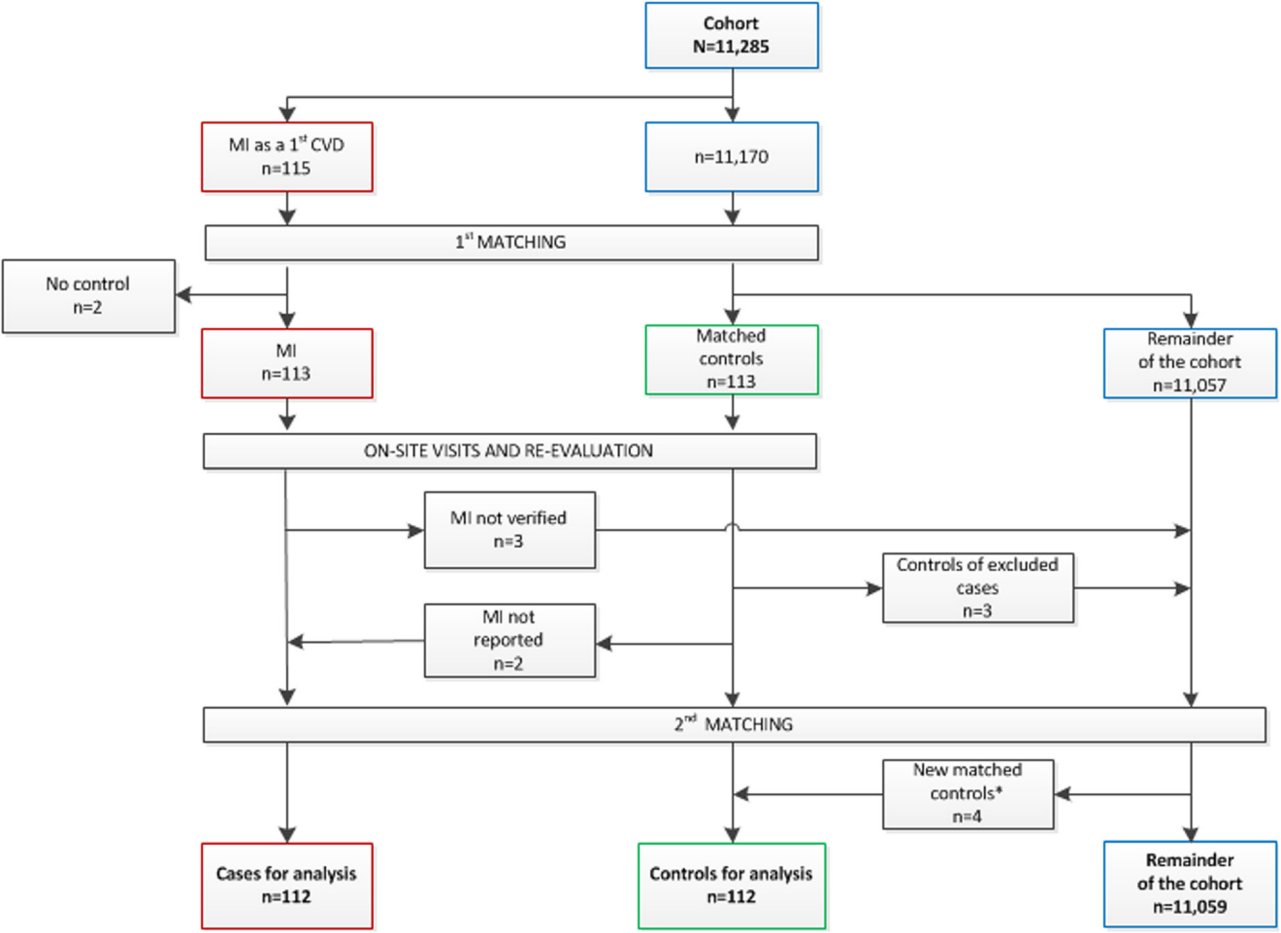
Flow chart for patient selection. *Because two controls turned out to be cases, four new controls had to be found in the second matching. *MI* myocardial infarction, *CVD* cardiovascular disease

Between 1 May 2001 and 31 October 2013, a total of 11,285 patients were enrolled into the RABBIT register (Fig. 1). Within that period of time, rheumatologists reported 115 MIs as a first CV event. Due to the exact matching algorithm matching controls were not found for four male cases (aged 62, 64, 68 and 76 years) with heart failure as a comorbidity. They were matched to controls with heart failure but were allowed to differ from their corresponding case in no more than two comorbidities (differences in hypertension in one pair, in CHD in two pairs, in previous cerebrovascular events in two pairs and in hyperlipoproteinaemia in one pair). For two further male MI cases no appropriate controls were found, due to their comorbidity status. These two patients were excluded. Similarly, patients with non-confirmed MI (n = 3) were excluded. During on-site visits, patients with non-reported MI (n = 2) were identified and included with a matching control. In total 112 eligible case–control pairs remained for the analyses (Fig. 1).

### Characteristics of matched pairs and the remainder of the cohort at baseline

Case–control pairs differed significantly from other patients in the RABBIT cohort in all matching parameters except for previous cerebrovascular events. In addition, cases differed from the cohort in most of the non-matching criteria (Table 1). Cases had significantly higher CRP, ESR and DAS28, and more impaired physical function (FFbH) and comorbidities (diabetes and chronic lung or renal disease). Compared to the cohort, MI cases were more often treated with oral glucocorticoids (93.6 % vs. 79.6 %, *p* < 0.01).

**Table 1:** Baseline characteristics of cases, controls and the remainder of the RABBIT cohort

	Cases n = 112	Controls n = 112	Remainder of the cohort^a^ n = 11,059
Matching criteria			
Sex, male	48 (42.9)	48 (42.9)	2536 (22.9)^‡^
Age, years, mean (SD)	63.7 (9.1)	63.7 (9.1)	55.9 (12.5)^‡^
Hypertension	67 (60.4)	68 (60.7)	4102 (37.1)^‡^
Coronary heart disease	28 (25.2)	26 (23.2)	622 (5.6)^‡^
Heart failure	7 (6.3)	7 (6.3)	242 (2.2)^‡^
Previous cerebrovascular event	0 (0)	2 (1.8)	146 (1.3)
Hyperlipoproteinemia	19 (17.1)	18 (16.1)	869 (7.9)^‡^
Time to MI/index date, month, mean (SD)	31.0 (24.9)	29.5 (23.9)	NA
Unmatched criteria			
Observation time, months, mean (SD)	52.6 (28.6)	60.2 (28.0)^†^	44.4 (32.7)^‡^
Disease duration, years, mean (SD)	11.4 (10.6)	11.4 (9.4)	10.0 (9.1)
Rheumatoid factor positive	83 (74.1)	85 (75.9)	7942 (72.1)
CRP, mg/L, mean (SD)	23.5 (27.0)	16.5 (22.1)^†^	18.4 (26.6)^‡^
ESR, mm/h, mean (SD)	39.2 (28.9)	30.7 (20.6)^†^	31.3 (23.0)^‡^
DAS28, mean (SD)	5.6 (1.3)	5.5 (1.3)	5.2 (1.3)^‡^
FFbH, mean (SD)	53.1 (24.8)	58.4 (23.3)	63.0 (23.3)^‡^
Smoking, current	25 (22.3)	19 (17.0)	2355 (21.3)
Smoking, former	35 (31.3)	24 (21.4)	2589 (23.4)
Smoking, never	35 (31.3)	61 (54.5)	4698 (42.5)
Smoking, unknown	17 (15.2)	8 (7.1)	1417 (12.8)
BMI, mean (SD)	28.1 (5.6)	26.7 (4.0)^†^	26.6 (5.3)^‡^
BMI ≥30 kg/m^2^	34 (30.4)	19 (17.0)^†^	2514 (22.7)
Diabetes mellitus	26 (23.4)	14 (12.5)^†^	1075 (9.7)^‡^
Chronic renal disease	11 (9.9)	8 (7.1)	397 (3.6)^‡^
COPD	12 (10.8)	13 (11.6)	495 (4.5)^‡^
No. of previous csDMARDs, mean (SD)	2.6 (1.4)	2.8 (1.5)	2.4 (1.3)
No. of previous bDMARDs, mean (SD)	0.5 (1.0)	0.4 (0.9)	0.3 (0.7)
Oral glucocorticoids	103 (93.6)	87 (77.7)^†^	8788 (79.6)^‡^
Glucocorticoids, <5 mg/day	12 (10.9)	29 (25.9)	2981 (27.0)
Glucocorticoids, 5–10 mg/day	64 (58.2)	46 (41.1)	4997 (45.3)^‡^
Glucocorticoids, ≥10 mg/day	34 (30.9)	37 (33.0)	3048 (27.6)^‡^
Non-selective NSAIDs	47 (42.0)	39 (34.8)	4260 (38.5)
COX-2 inhibitors	17 (15.2)	23 (20.5)	1699 (15.4)
Any NSAIDs	62 (55.4)	62 (55.4)	5895 (53.3)
No CV treatment^b^	27/75 (36.0)	13/75 (17.3)^†^	967/4584 (21.1)^‡^

Values are numbers of patients (%) unless otherwise specified. ^a^Patients without myocardial infarction (*MI*) at follow-up and patients who were not matched controls. ^b^No cardiovascular (*CV*) treatment: one or more of the reported cardiovascular disease (*CVD*) at baseline (hypertension, coronary heart disease, heart failure or hyperlipoproteinaemia) is not reported as being treated

*BMI* body mass index, *SD* standard deviation, *CRP* C-reactive protein, *ESR* erythrocyte sedimentation rate, *DAS28* disease activity score based on 28 joints, *FFbH* Hannover Functional Status Questionnaire, *COPD* chronic obstructive pulmonary disease, *csDMARD* conventional synthetic disease-modifying antirheumatic drug, *bDMARD* biologic DMARD, *NSAID* nonsteroidal anti-inflammatory drug, *COX-2* cyclooxygenase-2, *NA* not applicable. †*P* < 0.05 for comparison with cases (paired *t* test or Mc Nemar´s test). ‡*P* < 0.05 for comparison with cases (unpaired *t* test or chi-squared test)

The enrolment therapies (TNFi, other bDMARDs or csDMARDs) were similarly distributed between cases, controls and the cohort, with 45.5 % of cases on TNFi, 21.4 % on other bDMARDs and 33.1 % on csDMARDs; the corresponding figures among controls were 42.9 %, 21.4 % and 35.7 %, and among the cohort, 50.8 %, 16.2 % and 33.0 %, respectively.

Despite good agreement in the matching criteria, there were significant differences between cases and controls in CRP and ESR, obesity (body mass index (BMI) ≥30 kg/m^2^), diabetes and use of glucocorticoids. Importantly, among 75 case–control pairs with at least one baseline CV comorbidity, those patients who developed an MI during follow-up (cases) were significantly less likely to receive medical treatment for their CV comorbidity than their corresponding controls (36 % vs. 17 %, *p* < 0.01, Table 1).

### Treatment with DMARDs during follow-up

The number of different DMARD episodes was significantly higher in patients with MI than in matched controls, with one episode in 51 cases (45.5 %), two episodes in 30 cases (26.8 %) and ≥3 episodes in 31 cases (27.7 %); in controls the corresponding figures were 77 (68.8 %), 19 (16.9 %) and 16 (14.3 %), respectively (*p* < 0.01, paired *t* test).

Persistence with bDMARD enrolment therapy was significantly lower in cases compared to controls (*p* < 0.01, log rank test). In 50 pairs who started simultaneously with a bDMARD, 54.9 % (95 % CI 38.5; 68.5) of the cases compared to 76.5 % (95 % CI 60.4; 86.7) of the controls were still on the enrolment therapy at month 12. In addition, prior to the MI/index date the number of treatment switches (between different DMARDs) was about 53 % higher in cases (Poisson regression 1.53, 95 % CI 1.04; 2.27) than in respective controls. The median duration of a DMARD episode was 7 months in cases (IQR 4–17) and 13 months in controls (IQR 6–23).

### Disease activity and inflammation during follow-up

**Fig. 2:**
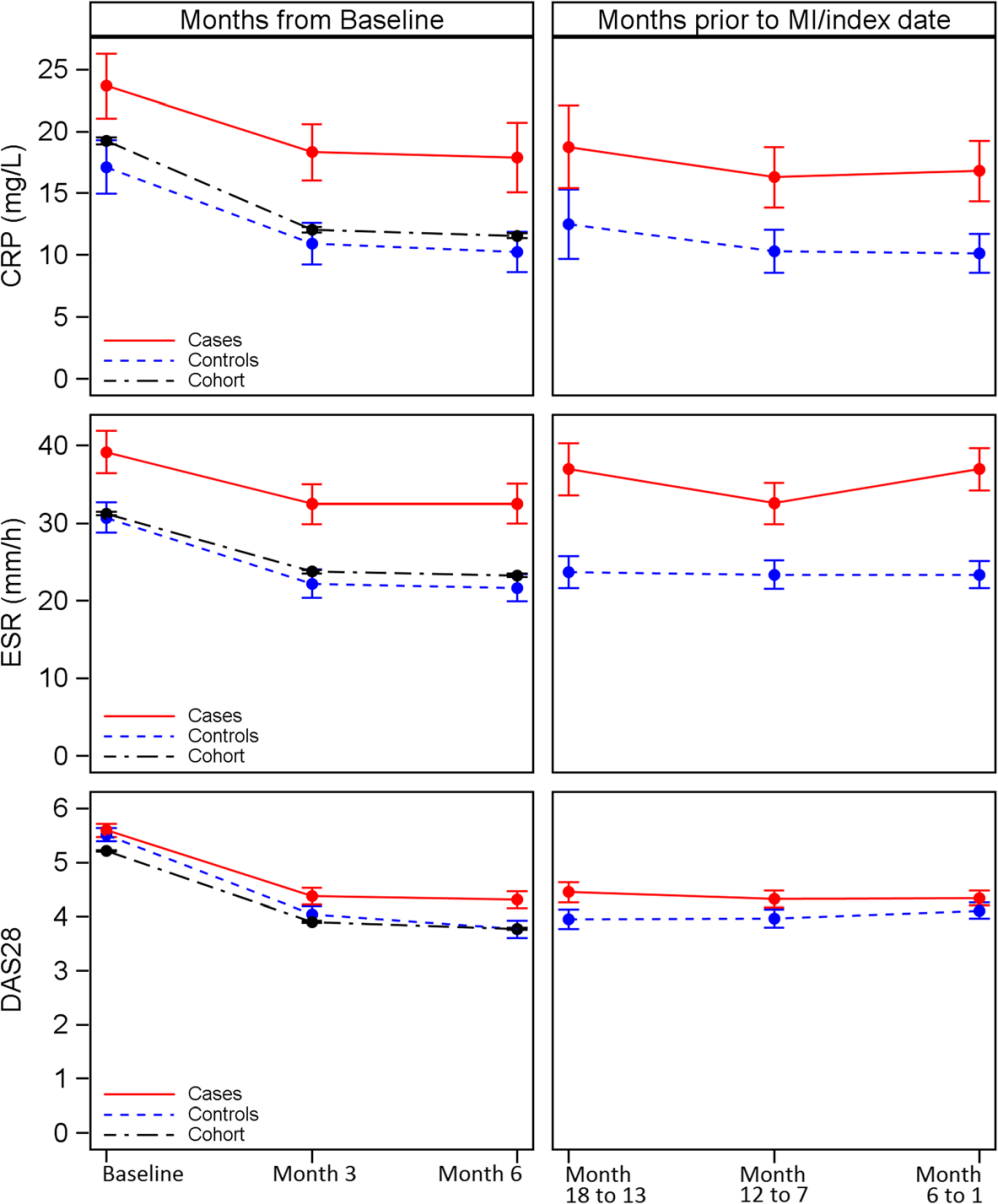
Development of mean C-reactive protein (*CRP*, mg/L), mean erythrocyte sedimentation rate (*ESR*, mm/h) and mean disease activity score based on 28 joints (*DAS28*) (all presented with *error bars*) at baseline, month 3 and month 6 in cases, matched controls and the remainder of the RABBIT cohort (*left*) and 18 months prior to the myocardial infarction (*MI*)/index date in cases and matched controls (*right*)

During the first 6 months from baseline, the inflammation markers CRP and ESR were significantly elevated in MI cases (Fig. 2, left; Table 2). In contrast, matched controls achieved similar improvements to the rest of the cohort (Table 2).

**Table 2:** Development of inflammation and disease activity in cases, controls and the remainder of the RABBIT cohort stratified by enrolment therapy

	Treatment at baseline	Number	Mean at baseline (95 % CI)	Mean at month 3 (95 % CI)	Mean at month 6 (95 % CI)
CRP (mg/L)					
Cases	csDMARD	37	21.6 (12.4; 30.8)	16.3 (9.0; 23.6)	14.3 (7.3; 21.2)
	bDMARD	75	24.4 (18.2; 30.6)	19.5 (14.5; 24.5)	19.4 (13.5; 25.4)
Controls	csDMARD	40	10.2 (6.1; 14.3)	9.8 (5.1; 14.5)	8.0 (4.7; 11.3)
	bDMARD	72	20.0 (14.1; 25.9)	11.4 (7.4; 15.4)	11.0 (6.9; 15.0)
Cohort remainder	csDMARD	3656	14.1 (13.5; 14.8)	11.4 (10.8; 11.9)	11.0 (10.4; 11.5)
	bDMARD	7403	20.75 (20.1; 21.4)	12.9 (12.4; 13.3)	12.7 (12.2; 13.1)
ESR (mm/h)					
Cases	csDMARD	37	30.4 (23.9; 36.9)	27.7 (20.9; 34.5)	31.4 (23.8; 39.0)
	bDMARD	75	43.2 (35.9; 50.4)	36.8 (30.5; 43.2)	34.7 (28.7; 40.7)
Controls	csDMARD	40	26.8 (20.2; 33.4)	22.4 (16.5; 28.2)	19.4 (13.1; 25.7)
	bDMARD	72	32.8 (28.1; 37.5)	22.7 (18.4; 27.1)	22.6 (18.8; 26.4)
Cohort remainder	csDMARD	3656	27.4 (26.7; 28.0)	23.9 (23.3; 24.5)	23.5 (22.8; 24.2)
	bDMARD	7403	33.3 (32.7; 33.8)	24.4 (24.0; 24.9)	24.4 (24.0; 25.0)
DAS28					
Cases	csDMARD	37	5.2 (4.8; 5.5)	4.0 (3.5; 4.4)	3.9 (3.4; 4.3)
	bDMARD	75	5.8 (5.5; 6.1)	4.6 (4.2; 5.0)	4.6 (4.2; 5.0)
Controls	csDMARD	40	4.8 (4.4; 5.3)	4.0 (3.6; 4.5)	3.5 (3.0; 4.0)
	bDMARD	72	5.9 (5.6; 6.1)	4.1 (3.7; 4.4)	3.9 (3.6; 4.3)
Cohort remainder	csDMARD	3656	4.8 (4.7; 4.8)	3.8 (3.7; 3.8)	3.7 (3.6; 3.7)
	bDMARD	7403	5.4 (5.4; 5.5)	4.0 (3.9; 4.0)	3.9 (3.8; 3.9)

Mean values are averaged over five imputations and CI were corrected for the imputation variance

*CI* confidence interval, *CRP* C-reactive protein, *ESR* erythrocyte sedimentation rate, *DAS28* disease activity score based on 28 joints, *bDMARD* biologic disease-modifying antirheumatic drug, *csDMARD* conventional synthetic DMARD

The distinct differences between cases and controls were still observed during the last 6 months (Table 3) and the last 18 months prior to the MI/index date (Fig. 2, right). Notably, no differences were found in DAS28.

**Table 3:** Characteristics of cases and matched controls within six months before the MI/index date

	Cases n = 105	Controls n = 105	*P* value
CRP, mg/L, mean (SD)	17.6 (25.0)	10.4 (14.6)	0.011
ESR, mm/h, mean (SD)	36.1 (26.5)	22.6 (16.2)	<0.001
DAS28, mean (SD)	4.3 (1.4)	4.0 (1.5)	0.22
Tender joint count, mean (SD)	4.2 (5.0)	4.4 (5.6)	0.71
Swollen joint count, mean (SD)	3.8 (5.0)	4.6 (5.4)	0.17
NRS patient global health 0–10, mean (SD)	5.1 (2.2)	4.9 (2.0)	0.41
FFbH, mean (SD)	58.7 (27.1)	61.0 (24.2)	0.32
TNFi	50 (47.6 %)	55 (52.4 %)	0.41
Other bDMARDs	21 (20.0 %)	23 (21.9 %)	0.66
csDMARDs only	33 (31.4 %)	23 (21.9 %)	0.11
Glucocorticoids, <5 mg/day	44 (41.9 %)	62 (59.6 %)	
Glucocorticoids, 5–10 mg/day	45 (42.9 %)	34 (32.7 %)	0.008
Glucocorticoids, ≥10 mg/day	16 (15.2 %)	8 (7.7 %)	
Non-selective NSAIDs	60 (57.1 %)	62 (59.0 %)	0.77
COX-2 inhibitors	28 (26.7 %)	36 (34.3 %)	0.19
Any NSAID	72 (68.6 %)	78 (74.3 %)	0.33

Case–control pairs with missing C-reactive protein (*CRP*) values were not included in this analysis. Data represent averages of all reported values within 6 months before the myocardial infarction (*MI*)/index date. All values are numbers of patients (%) unless otherwise specified. Tumour necrosis factor inhibitors (*TNFi*), other biologic disease-modifying antirheumatic drugs (*bDMARDs*) and conventional synthetic DMARDS (*csDMARDs*) were counted if the patient received at least one dose of the drug within 6 months before the MI/index date. For nonsteroidal anti-inflammatory drugs (*NSAIDs*) and cyclooxygenase-2 (*COX-2*) inhibitors, data represent use in the 24 months before the MI/index date

*SD* standard deviation, *ESR* erythrocyte sedimentation rate, *DAS28* disease activity score based on 28 joints, *NRS* numeric rating scale, *FFbH* Hannover Functional Status Questionnaire

The exclusion of patients with underlying CHD at baseline did not alter the course of inflammation and disease activity (Additional file 1).

### Evaluation of risk factors for MI

In the univariate logistic comparison of cases and controls there was an increased risk of MI with an increase of CRP per 5 mg/L (OR 1.13 (95 % CI 1.02; 1.22) based on values obtained within 6 months prior to the MI/index date). The association with risk of MI was stronger with log-transformed CRP (1.75 (1.24; 2.46)). Similarly, averaged and log-transformed CRP over the total observation time was significantly associated with risk of MI (1.75 (1.26; 2.43)). Other significant predictors were absence of baseline CV treatment (3.60 (1.28; 12.40)) and glucocorticoids at a dosage of 5–10 mg/day (1.97 (0.98; 4.11) and ≥10 mg/day (3.02 (1.11; 8.25) vs. glucocorticoids <5 mg/day). Previous or current smoking (3.15 (1.47; 7.34)) and unknown smoking status (2.68 (1.06; 7.30)) were significant risk factors compared to not smoking. There was no significant association between increased risk of MI and TNFi or other bDMARD treatment (reference csDMARDs). The adjusted multiple conditional logistic regression revealed no association between risk of MI and treatment with TNFi or other bDMARDs, and no significantly higher risk with glucocorticoid treatment of 5–10 mg/day or ≥10 mg/day (Table 4). There was a strong association between log CRP and MI, but not between raw CRP values and MI, confirming the expected non-linear association. Smoking was confirmed as another significant risk factor.

**Table 4:** Multivariate odds ratios for the risk of MI in the nested case–control analysis

	All matched case–control pairs				Subset of cases and controls without CHD			
	I		II		I		II	
	OR	(95 % CI)	OR	(95 % CI)	OR	(95 % CI)	OR	(95 % CI)
Log CRP, prior MI^a^	1.58	(1.07; 2.33)			1.60	(1.04; 2.46)		
Log CRP, total observation^a^			1.47	(1.00; 2.16)			1.44	(0.94; 2.19)
csDMARDs only	Ref.		Ref.		Ref.		Ref.	
TNFi	0.96	(0.41; 2.22)	0.91	(0.40; 2.10)	1.24	(0.49; 3.16)	1.22	(0.49; 3.05)
Other bDMARDs	1.13	(0.34; 3.71)	0.85	(0.27; 2.72)	0.86	(0.19; 3.84)	0.53	(0.13; 2.18)
Glucocorticoids <5 mg/day	Ref.		Ref.		Ref.		Ref.	
5–10 mg/day	1.33	(0.61; 2.89)	1.22	(0.56; 2.68)	1.42	(0.59; 3.43)	1.32	(0.55; 3.17)
≥10 mg/day	2.17	(0.69; 6.81)	1.83	(0.57; 5.85)	2.48	(0.72; 8.59)	2.18	(0.61; 7.79)
No CV treatment^b^	2.76	(0.91; 8.32)	2.66	(0.88; 8.00)	2.42	(0.55; 10.77)	2.66	(0.60; 11.72)
Smoking never	Ref.		Ref.		Ref.		Ref.	
Smoking ever	3.33	(1.45; 7.63)	2.93	(1.29; 6.66)	2.13	(0.85; 5.32)	2.03	(0.69; 5.93)
Smoking status unknown	2.15	(0.82; 5.66)	2.12	(0.80; 5.65)	1.74	(0.62; 4.88)	1.84	(0.65; 5.21)
Diabetes	2.08	(0.84; 5.18)	2.32	(0.94; 5.71)	1.95	(0.68; 5.63)	1.92	(0.67; 5.51)

Case–control pairs with missing C-reactive protein (*CRP*) values were not considered in this analysis. ^a^All CRP values were log-transformed. Analysis I: CRP values of the last 6 months prior to the myocardial infarction (*MI*)/index date, Analysis II: averaged CRP values from baseline until the MI/index date. ^b^No CV treatment: one or more of the types of reported cardiovascular disease (CVD) at baseline (hypertension, coronary heart disease, heart failure and hyperlipoproteinaemia) is not reported as being treated. Baseline information was used for no CV treatment, smoking and diabetes. All other treatments are values within 6 months before the MI/index date

*OR* odds ratio, *CI* confidence interval, *CHD* coronary heart disease, *bDMARD* biologic disease-modifying anti-rheumatic drug, *csDMARD* conventional synthetic DMARD, *TNFi* tumour necrosis factor inhibitor

The risk imposed by elevated CRP remained in a sub-analysis of patients without CHD at baseline, although this was no longer statistically significant for values averaged over the complete observation period (until the MI/index date).

## Discussion

We investigated the risk of MI in a large observational cohort study of 11,285 patients with established RA. An in-depth comparison of patients who developed MI with the remaining cohort revealed wide-ranging differences in patient characteristics and exemplified the need for an appropriate study design beyond covariate adjustment. To account for these differences we applied a nested case–control design using an extensive matching algorithm that enabled us to link homogeneous case–control pairs.

In a setting that controlled for traditional risk factors, we found that inflammation and smoking were significantly associated with the risk of MI in patients with RA. At baseline, during the first 6 months of follow-up and, more importantly, prior to the MI/index date (Fig. 2), there was a distinct difference in CRP and ESR levels between cases and controls. The significant differences in CRP values remained throughout the period of observation. This result confirms recent findings of others who report that the risk of MI is highest for patients with RA who have high CRP [15]. Similar to the results of the Emerging Risk Factors Collaboration [27], our data suggest a nonlinear increase in risk of MI with rising CRP. Compared with the significantly increased risk of MI with high sensitivity CRP values above 1 mg/L among the general population [28], our data suggest that the complete suppression of systemic inflammation in RA may reduce the risk of MI.

Others have reported lower risk of MI in TNFi responders vs. TNFi non-responders [29, 30]. However, based on the DAS28, patients can respond even when CRP remains high [31], which is in line with our data. Patients with MI presented with similar values of the DAS28 prior to the MI/index date compared to control patients, but with significantly elevated CRP and ESR. We conclude that the evaluation of RA disease activity solely based on the DAS28 may not be sufficient to predict risk of MI. The assessment should also comprise the inflammatory marker CRP, particularly in patients with present CVD or at increased risk of CVD.

We identified comparable treatment with DMARDs in patients who developed MI and in the matched controls at baseline only. During follow-up there were significant differences: rheumatologists switched the bDMARD treatment in cases significantly more often than in respective controls, which indicates continuous attempts to adapt the DMARD treatment. Nevertheless, switches remained ineffective in reducing CRP (Fig. 2). This result suggests that the available RA treatment options for these patients were insufficient. New biologic drugs with alternative targets have been available since 2007. A recent meta-analysis discussed IL-6 inhibition as a possible treatment target to prevent CVD [10]. This may be appropriate for patients not responding to other bDMARDs and with high average CRP. Due to the small number of tocilizumab episodes (13 of 242 cases (5.4 %) and 9 of 184 controls (4.9 %)), we could not study the impact of this treatment separately.

There are conflicting results from previous studies regarding the influence of glucocorticoid treatment on the risk of CVD. Some studies report a risk associated with higher doses of glucocorticoids [32–36]. As expected, in the univariate analysis we observed stronger association between prednisone dose and MI risk than in the multivariate analyses after adjustment for average CRP. These results suggest that the harmful effects of glucocorticoids reported by others are likely partly a result of patient channelling: patients who did not respond to the primary treatment with bDMARDs were consequently treated with glucocorticoids in higher doses. In this matter, concomitant glucocorticoids were used by rheumatologists as a kind of rescue therapy. We observed that the risk remained with glucocorticoid use ≥10 mg/day, but this was not statistically significant and needs to be investigated further in studies with sample sizes larger than ours.

An obvious but rather unexpected risk factor was detected in our data: in patients with a future MI, pre-existing CV comorbidities were less frequently treated than in the corresponding control patients. This suggests that insufficient consideration of CV risk in patients with known CV comorbidities is a further risk factor for MI. There seems to be a gap between the knowledge about CV risk in RA, respective recommendations [20, 37] and the daily management of patients. Our findings confirm suboptimal risk management of CVD [38, 39]. One of the weaknesses of this study is the uncertainty about the first-ever MI. In some of the patients the information about the first MI was subsumed in the comorbidity of CHD at baseline. Therefore, we performed a sub-analysis in patients without reported CHD at baseline. We calculated consistent estimates. A strength of this study is the comprehensive on-site validation process, which revealed low numbers of underreported MI in patients with elevated risk of a CV event. However, on-site validation was stopped after reviewing 75 case–control pairs, as very little additional information was obtained on laboratory parameters or CV treatment. The size of the nested case–control study was too small to estimate the separate effects of abatacept, rituximab and tocilizumab [40, 41], or those of methotrexate, leflunomide or other csDMARDs. The total amount of missing data during follow-up was low (<10 %). Nevertheless, we applied multiple imputations but the impact on estimates was statistically and clinically insignificant when compared to an analysis of pairs with observed data only.

## Conclusion

In conclusion, our results underline the importance of a treat-to-target approach, which has to take the global disease activity and CRP into account. As inflammation is the link to CVD, we consider CRP the most reliable marker to assess the risk of MI. For many patients it seems less important by which DMARD treatment (TNFi, other bDMARD or csDMARD) the treatment target is reached. In some patients however, the available treatment options were insufficient or insufficiently used. This adds to the evidence indicating the necessity of tight disease control and adequate treatment of comorbidities.

## Abbreviations

bDMARD, biologic disease-modifying antirheumatic drug; BMI, body mass index; CHD, coronary heart disease; CI, confidence interval; COPD, chronic obstructive pulmonary disease; COX-2, cyclooxygenase-2; CRP, C-reactive protein; csDMARD, conventional synthetic disease-modifying antirheumatic drug; CV, cardiovascular; CVD, cardiovascular disease; DAS28, disease activity score based on 28 joints; ESR, erythrocyte sedimentation rate; EULAR, European League Against Rheumatism; FFbH, Hannover Functional Status Questionnaire; IL, interleukin; IQR, interquartile range; logCRP, log-transformed C-reactive protein; MI, myocardial infarction; NRS, numeric rating scale; NSAID, nonsteroidal anti-inflammatory drug; RA, rheumatoid arthritis; RABBIT, Rheumatoid Arthritis Observation of Biologic Therapy; SD, standard deviation; SNP, single nucleotide polymorphism; TNF, tumour necrosis factor

## Additional file

Additional file 1: Figure S1. Course of RA disease represented by CRP, ESR and DAS28, and restricted to patients without coronary heart disease at baseline. (DOCX 128 kb)
