# Supplementary material for: Impact of disease activity and treatment of comorbidities on the risk of myocardial infarction in rheumatoid arthritis
Source: Arthritis Res Ther. 2016 Aug 5;18:183. doi: 10.1186/s13075-016-1077-z (PMC4975917; doi:10.1186/s13075-016-1077-z)
Supplement: Additional file 1: Figure S1. — Course of RA disease represented by CRP, ESR and DAS28, and restricted to patients without coronary heart disease at baseline. (DOCX 128 kb) [file 13075_2016_1077_MOESM1_ESM.docx]

**Figure S1:** Course of RA disease represented by CRP, ESR and DAS28, and restricted to patients without coronary heart disease at baseline.


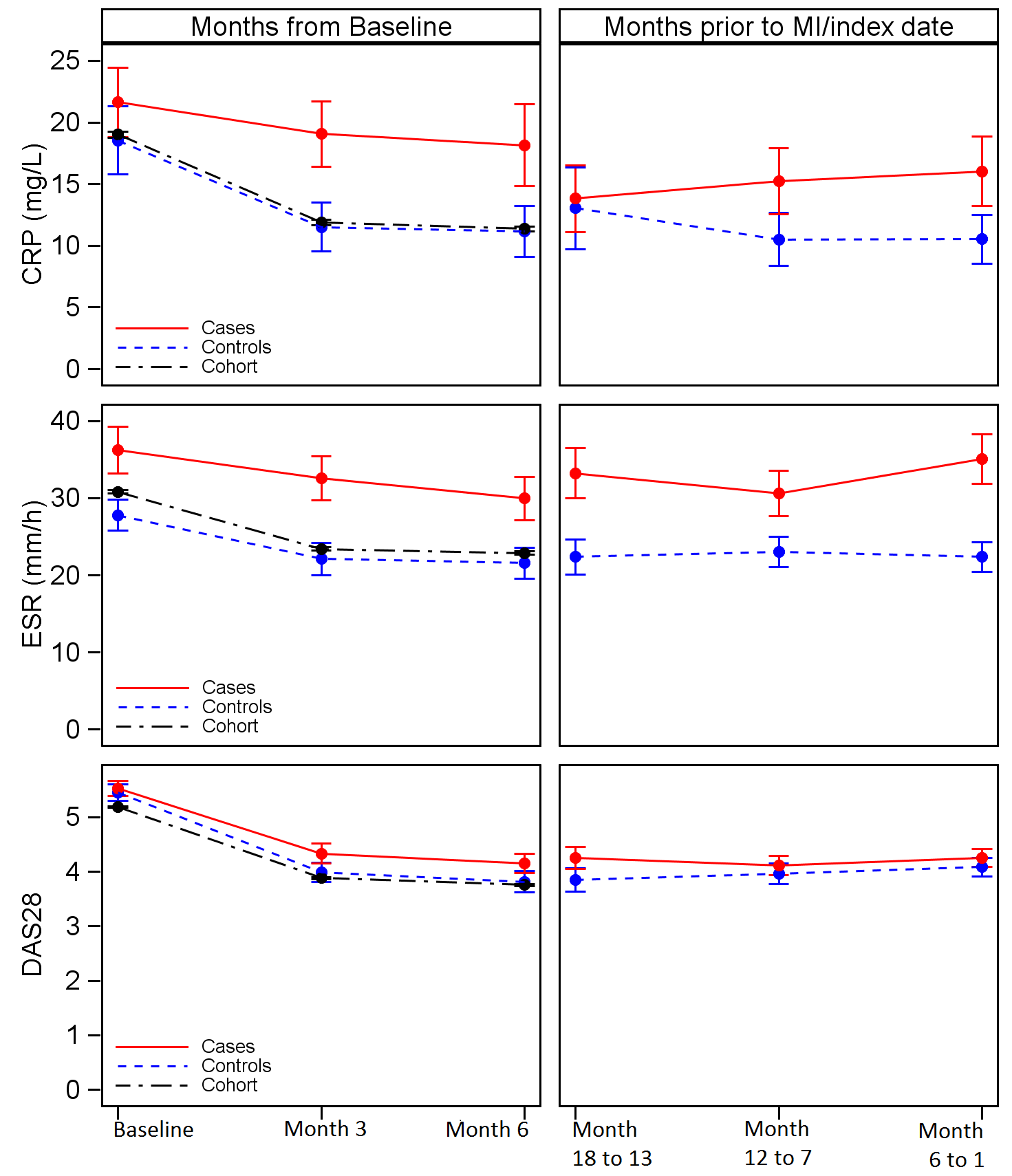


*Development of mean CRP (in mg/L), mean ESR (in mm/h) and mean DAS28 (all presented with error bars) at baseline, month 3 and month 6 in cases, matched controls and the remainder of the RABBIT cohort (left) and 18 months prior to the MI/index date in cases and matched controls (right).*
